# Dr. Paris, on Animal Heat

**Published:** 1809-01

**Authors:** John Ayrton Paris

**Affiliations:** Westminster


### To Dr. BRADLEY.

Dear Sir,

THAT Dr. Crawford's theory for the explanation of the phænomena of animal heat possesses much ingenuity, and that the experiments by which it is supported are correct and imposing, every physiologist must admit; but that it has also many assailable points, its most zealous partisan will not deny; and here it becomes necessary to offer a short analysis of the doctrine I propose to modify, although the subject must be well known to most of the readers of this article.

He assumes as the basis of his theory that arterial blood becomes venous, and loses its energies, by combining with *hydro-carbon* in the extreme vessels; a product, as he supposes, of the putrefactive action that useless and decayed parts undergo. The existence however of such a compound is neither proved by experiment nor induction; it is not the office of the veins but of the lymphatics to remove broken down and useless structure, in addition to which, carbon and hydrogen are not the principal products of putrefaction; and it appears from the experiments of John Hunter, that arterial gains all the properties of venous blood by rest, which proves most satisfactorily that the transition depends on some internal change in the constitution of the blood, and not on the addition of any foreign matter; nor are the subsequent parts of Crawford's theory less exceptionable; he supposes that the blood thus saturated with *hydro-carbon* returns to the lungs, where, by the affinity of oxygen to the constituents of this compound, water and carbonic acid are formed, and that the blood is thus released from its injurious contamination; and as the capacities of these new compounds for caloric are less than those of their constituents, he endeavours to demonstrate that an evolution of caloric takes place, which would be sufficiently great

 to to raise a bar of iron to a red heat, did not the blood at the same instant acquire a proportionate increase of capacity, which by gradually diminishing as it becomes venous, dispenses its caloric with uniformity to every part of the body: it appears however by no means evident, that water and carbonic acid are generated in the lungs by the chemical union of their constituents. Dr. Bostock supposes the watery vapour that is expired to be derived from the evaporation of the fluid that lubricates the bronchiæ; I should be more willing however to explain its origin, by supposing that the relative quantity of water in the blood becomes greater by the expenditure of its other principles, in the formation of the different secretions, in the repairs of injured or decayed structure, and in the revival of muscular irritability. Neither is Dr. Crawford's opinion concerning the source of carbonic acid less questionable. Cuvier very correctly supposes that respiration is the principal agent in the process of sanguification, and as chyle differs from fibrine only in possessing less azot, and a greater proportion of carbon, such a theory will well explain the source from which the carbonic acid is derived, and the indispensable necessity of azot in our atmosphere.

It will appear therefore that the theory of Dr. Crawford is deduced from uncertain data; for if it be shewn that water is not generated in the lungs, it is deprived of its greatest support, and the existence of the principal source from which he conjectures animal heat to proceed is disproved. That the capacity of the arterial blood for caloric, is greater however than that of venous, is too evident to be doubted, and must therefore be acknowledged as one cause on which the heat of animals depends; but it is nevertheless insufficient to account for its copious production, some other source must be therefore investigated, which I hope I shall be able to ascribe, with some justice, to the process of secretion.

Whatever diversity of opinion may be maintained concerning the phenomena of secretion, it will be generally admitted, that the secreted fluids are new chemical compounds formed by the glands from the principles of the blood; if then it be demonstrated that the capacities of these compounds for caloric be less than that of the blood, we shall at once discover a considerable source of animal heat, which in conjunction with that arising from the different capacities of arterial and venous blood, will be adequate equate to the full illustration of its phænomena; to ascertain this fact I instituted the following experiments.

### Experiment I.

Temperature of the Laboratory 46° Fah.

| | | |
|---|---|---|
| Of urine 1 pint was heated to | 65° | Arithmetic mean |
| Of water . . . . . . . | 113 | 89. |
| Temperature that resulted . | 90 | |
| Escaped during the experiment | 2 | |
| True temperature . . . . . | 92 | |

From the above results it appears that the water has been deprived of 21° of heat, which has raised the urine 27°: from which it is evident that the capacity of urine is to that of water as 21 : 27 or as 7 : 9. Hence let $x$ represent the capacity of urine, and 1·000 that of water; then

$$7 : 9 :: x : 1{\cdot}000$$
$$9x = 7{\cdot}$$
$$\therefore x = \tfrac{7}{9} = {\cdot}7777;$$

whereas the capacity of arterial blood is 1·003.

### Experiment II.

To determine the capacity of Bile.

| | | |
|---|---|---|
| Of bile 1 pint was heated to | 86° | Arithmetic mean |
| Of water 1 pint . . . | 114 | 100. |
| Resulting temperature . | 100 | |
| Escaped . . . . . . . . | 2 | |
| True temperature . . . | 102 | |

In this experiment water has lost 12° of heat, which has raised the bile 16°, therefore the capacity of bile is to that of water, as 12 : 16, or as 3 : 4. Hence

$$\text{As } 3 : 4 :: x : 1{\cdot}000$$

$$4x = 3$$

$$x = \tfrac{3}{4} = {\cdot}75 = \text{capacity of bile;}$$

whereas the capacity of venous blood is ·8928.

Another example of the capacity of secreted fluids being less than that of the blood from which they are secreted, is exhibited in the table annexed to Dr. Crawford's work; he there shews, that the capacity of milk is only ·9999.

Hence by analogy we may conclude, that all the secreted fluids possess a capacity for caloric, less than that of of the blood, and that consequently the secreting process must be a considerable instrument in the production of animal heat.

The argument of Black will doubtless be urged in defence of Crawford's theory, that the temperature of an animal is always proportionate to the extent of its respiratory apparatus. To this I will reply, that nature observes the greatest uniformity in all her operations, and seems to have established an invariable relation between the different organs of animals; hence the structure of those endowed with extensive organs of respiration is more complicated, and consequently, the secretions are more numerous and perfect. If we descend in the scale of animated existence, we shall discover that the same relation is preserved; thus the secreting apparatus seems to consist only of simple tubes, in those animals whose organs of respiration are inconsiderable; and in the Zoophytes, by which no respirations appear to be performed, not the least vestige of any gland or secreting organ is discoverable.

Let us now consider whether the animal temperature varies in proportion to the increase or decrease of the secretions. The sum of the secretions is commonly the same; for if any single secretion suffers a change in quantity, we shall find the others varying so as to preserve the same general effect; thus, for instance, if the urine flows with greater abundance, the saliva or perspirable matter is secreted less copiously; if however a universal cause affects the body, so as to diminish all its secretions, we find the animal temperature sinks, as is exemplified by sleep, or the influence of the depressing passions.

I am, &c.

JOHN AYRTON PARIS.

*Westminster, December* 10, 1808.

